# Haiti in the Context of the Current Global Cholera Pandemic

**DOI:** 10.3201/eid1711.110849

**Published:** 2011-11

**Authors:** Edward T. Ryan

**Affiliations:** Massachusetts General Hospital, Boston, Massachusetts USA;; Harvard Medical School, Boston

**Keywords:** cholera, bacteria, Haiti, pandemic

Since the early 1800s, there have been 7 cholera pandemics, and 2011 marks not only the 1-year anniversary of the reappearance of cholera in Haiti but also the 50th anniversary of the onset of the current cholera pandemic that began in Indonesia in 1961. All previous pandemics lasted 5–25 years before burning out. However, the current pandemic has shown no evidence of abating. Cholera is a disease of impoverishment, displacement, and unrest, and the 2010–2011 Haiti and global cholera milestones are integrally related. In addition to Haiti, during the past 10 years, there have been major cholera epidemics in Zimbabwe, Kenya, Nigeria, Afghanistan, Iraq, Somalia, Angola, Guinea-Bissau, Sudan, South Africa, Malawi, Liberia, and Vietnam. Cholera is endemic to >50 countries, affects 3–5 million persons each year, and kills 100,000. Most of these cases never come to public or media attention, and many of them occur in areas where cholera is deeply entrenched and often affects children. In some areas of southern Asia, most residents will have serologic evidence of infection with *Vibrio cholerae* by their teenage years.

Why has this pandemic persisted for so long? The answer is that we do not know, but several factors seem to be major contributors to its longevity. First, the organism is different from the version microbiologically associated with previous pandemics. Previous pandemics for which we have data were caused by the classical *V. cholerae* O1 biotype, but the current pandemic is caused by the El Tor biotype. *V. cholerae* persists in aquatic reservoirs, and for unclear reasons, the El Tor biotype seems to have a distinct transmission or environmental survival advantage and has replaced the *V. cholerae* classical biotype worldwide. This advantage may translate into increased likelihood that *V. cholerae* will become endemic and persist in a local environment after its introduction into new areas. The El Tor biotype is also associated with more prolonged clinical outbreaks, often featuring multiple waves, and has the ability to cause mild disease or short-term asymptomatic passage once established in a population. These features contribute to the silent introduction of cholera into new areas, as unfortunately occurred this past year in Haiti.

During the current pandemic, the El Tor biotype has continued to evolve. In the early 1990s, this biotype mutated to a new serogroup, O139, and rapidly spread to several countries in Asia, joining O1 as a cause of epidemic cholera. Previous immunity to *V. cholerae* O1 provided no protection against O139. The number of cases caused by O139 then decreased, leaving the O1 El Tor biotype as the predominant cause of cholera, perhaps again underscoring some poorly understood survival or transmission advantage of this biotype. During the past 2 decades, the organism again evolved and became hybrid, keeping its El Tor biotype characteristics but incorporating classical biotype cholera toxin, a feature that may be contributing to high case-fatality rates associated with many recent cholera outbreaks. *V. cholerae* continues to evolve, and resistance to antimicrobial drugs is complicating treatment options in areas with limited resources.

However, changes in the organism only partly explain the complexity of our current pandemic situation. Cholera is a disease of the most impoversished, but it is first and foremost a disease affected by the global economy and transportation, initially spreading from its ancestral home in southern Asia along trading and commerce routes of the nascent global economy of the early 1800s. Although cholera spreads through global interactions, it paradoxically predominantly affects those most estranged from the benefits of globalization. In the long term, economic investment and civil stability will lead to the demise of cholera, but with ≈1 billion persons currently lacking safe water, and 2.6 billion currently lacking adequate sanitation, our current war with cholera will go on for decades.

Do we just grin and bear it, or is it time to change our response strategy? No one would argue that cholera response programs need to be based on case detection, appropriate fluid management, and provision of safe water and improved sanitation. However, is it time to integrate new tools? Previous response plans grew from previous experience: wild fire cholera epidemics that burned out quickly, ability of rehydration strategies to decrease case-fatality rates to <1%, and a problematic parenteral cholera vaccine. However, with the propensity of the El Tor biotype to cause prolonged and recurrent outbreaks, high likelihood of becoming endemic, ability to be carried asymptomatically, association with case-fatality rates of 2%–6% among patients who receive clinical care during complex emergencies, and availabilty of improved oral cholera vaccines, is it time to rethink our plans? Should vaccines be used more broadly?

Strong evidence would support use of cholera vaccines in disease-endemic settings, and an evolving body of evidence, largely from increased interest in cholera after its appearance in Haiti, suggests that cholera vaccines might be beneficial in reactive situations, i.e., after an outbreak has started. However, such use would first require additional field and cost-effectiveness evaluations and intricate planning and commitment. Would an international stockpile of vaccine be beneficial? Who would support and manage it? What would be the triggers for its use? How would its benefit be measured? Similarly, should there be wider or more specific use of antimicrobial drugs in the initial stages of a cholera outbreak with the goal of blunting transmission? Would this buy time? Would such distribution be not only useless, but also detrimental, accelerating the development of antimicrobial drug resistance? And why is it so hard to get clean water and adequate sanitation to those who need it most? What are the obstacles? How can we improve our track record?

Quite simply, we do not yet know the answers to many of these questions, but we should not only view the cholera epidemic in Haiti as a true catastrophe, which it is of immense proportions, but we should also view it as an opportunity. Will we use the hydra-headed reappearance of cholera in Haiti as an impetus to adapt and respond, learning from our successes and failures, or will we be ill-prepared when cholera appears in the next Zimbabwe, the next Afghanistan, the next Haiti? The next Haiti will be here sooner than we think.

